# Gene Losses during Human Origins

**DOI:** 10.1371/journal.pbio.0040052

**Published:** 2006-02-14

**Authors:** Xiaoxia Wang, Wendy E Grus, Jianzhi Zhang

**Affiliations:** **1**Department of Ecology and Evolutionary Biology, University of Michigan, Ann Arbor, Michigan, United States of America; University of BathUnited Kingdom

## Abstract

Pseudogenization is a widespread phenomenon in genome evolution, and it has been proposed to serve as an engine of evolutionary change, especially during human origins (the “less-is-more” hypothesis). However, there has been no comprehensive analysis of human-specific pseudogenes. Furthermore, it is unclear whether pseudogenization itself can be selectively favored and thus play an active role in human evolution. Here we conduct a comparative genomic analysis and a literature survey to identify 80 nonprocessed pseudogenes that were inactivated in the human lineage after its separation from the chimpanzee lineage. Many functions are involved among these genes, with chemoreception and immune response being outstandingly overrepresented, suggesting potential species-specific features in these aspects of human physiology. To explore the possibility of adaptive pseudogenization, we focus on CASPASE12, a cysteinyl aspartate proteinase participating in inflammatory and innate immune response to endotoxins. We provide population genetic evidence that the nearly complete fixation of a null allele at *CASPASE12* has been driven by positive selection, probably because the null allele confers protection from severe sepsis. We estimate that the selective advantage of the null allele is about 0.9% and the pseudogenization started shortly before the out-of-Africa migration of modern humans. Interestingly, two other genes related to sepsis were also pseudogenized in humans, possibly by selection. These adaptive gene losses might have occurred because of changes in our environment or genetic background that altered the threat from or response to sepsis. The identification and analysis of human-specific pseudogenes open the door for understanding the roles of gene losses in human origins, and the demonstration that gene loss itself can be adaptive supports and extends the “less-is-more” hypothesis.

## Abstract 2

The authors find that the human *CASPASE12* pseudogene has been positively selected for in humans. This adaptive gene loss confers protection from sepsis.

## Introduction

Although humans are highly similar to chimpanzees at the genomic sequence and protein sequence levels [1–6], the two species differ dramatically in many aspects of their biology such as bipedalism, brain size, language/speech capability, and susceptibility to the human immunodeficiency virus (HIV)/simian immunodeficiency virus. With rapid progress in human genetics, comparative genomics, and molecular evolution, the genetic basis of these differences has begun to be unraveled. For example, the conserved transcriptional factor FOXP2 is required for speech development in humans [7], and it experienced two adaptive amino acid replacements in hominin evolution, suggesting that these two substitutions were at least partially responsible for the emergence of human speech and language [8,9]. Compared to such amino acid replacements, gene gains and losses are more dramatic genetic changes [10–14]. In particular, gene loss, or pseudogenization, leads to immediate loss of gene function, which probably affects organisms to a greater extent than do most amino acid replacements. A number of genes are known to have been lost in the human lineage since its divergence from the chimpanzee lineage [15–25]. Recently, Olson [11] and Olson and Varki [12] proposed the “less-is-more” hypothesis, suggesting that gene loss may serve as an engine of evolutionary change. This hypothesis is particularly intriguing for human evolution, as several human gene losses have been proposed to provide opportunities for adaptations and be responsible for human-specific phenotypes. For example, the pseudogenization of the sarcomeric myosin gene masticatory myosin heavy chain 16 *(MYH16)* at the time of the emergence of the genus *Homo* is thought to be responsible for the marked size reduction in hominin masticatory muscles, which may have allowed the brain size expansion [23] (but see [25]). In another example, the human-specific inactivation of the gene encoding the enzyme CMP-*N*-acetylneuraminic acid hydroxylase (CMAH) led to the deficiency of the mammalian common sialic acid Neu5Gc (*N*-glycolylneuraminic acid) on the human cell surface [19]. This inactivation was due to an Alu-mediated sequence replacement [26] that occurred about 2.7 million years ago [27] and may have had several important consequences to human biology and evolution [28].

It is thus interesting to systematically identify and analyze all human-specific gene losses. Here, a human-specific gene loss refers to a loss that occurred in the human lineage after the human-chimpanzee divergence; the gene may be lost independently in other species (except the chimpanzee). Two attempts to identify human-specific pseudogenes [29,30] have been made recently using comparative genomic approaches. However, the first analysis was limited by its comparison between genome sequences of humans and rodents, instead of chimpanzees [29]. The second analysis compared human mRNA with the chimpanzee genome sequence, missing all nontranscribed human pseudogenes [30]. As a result, these two studies identified only six and nine cases, respectively [29,30], and the majority of them are not even true human-specific pseudogenes due to the limitations of their methodologies (see below). Moreover, all human gene losses known to date presumably occurred by random fixations of null alleles at dispensable loci. There has been no demonstration of positive selection driving the loss of a human gene, although the loss may have subsequently allowed future adaptations. Such passive pseudogenization incidences are not themselves adaptations. In this work, we explore which types of genes have been lost in recent human evolution and determine if there is evidence for adaptive loss of human-specific pseudogenes. First, we identify human-specific gene losses by comparing human nonprocessed pseudogenes with the chimpanzee genome sequence. These human-specific pseudogenes were formed in the last 6 to 7 million years after the separation of humans and chimpanzees [31]. However, because positive selection for null alleles cannot be detected by comparing humans and chimpanzees, we rely on human population genetic data, which may retain signatures of selective sweeps for at most 200,000 years [32]. That is, such evidence is best sought among very recent pseudogenizations. Using this strategy, we provide evidence that the nearly complete fixation of a null allele at *CASPASE12 (CASP12)* [6,33,34] has been driven by positive selection, probably because the allele confers lowered susceptibility to severe sepsis.

## Results/Discussion

### Identification of Human-Specific Pseudogenes

The human genome has an abundance of pseudogenes [35,36], but the majority of them are processed pseudogenes [35,36], which are DNA sequences reverse-transcribed from RNA and randomly inserted into the genome. Although some processed pseudogenes may become functional genes fortuitously [37,38], the majority lack necessary regulatory elements or complete coding regions and are dead-on-arrival. Hence, most processed pseudogenes have never been functional. Consequently, these pseudogenizations should not have affected the organisms. In contrast, nonprocessed pseudogenes were once functional genes that now have their coding sequences interrupted. However, many nonprocessed pseudogenes are formed soon after gene duplication due to genetic redundancy [10]. In such cases, pseudogenizations are unlikely to have functional consequences either. We thus focus on human-specific nonprocessed pseudogenes but do not consider those resulting from human-specific gene duplicates.

**Figure 1:**
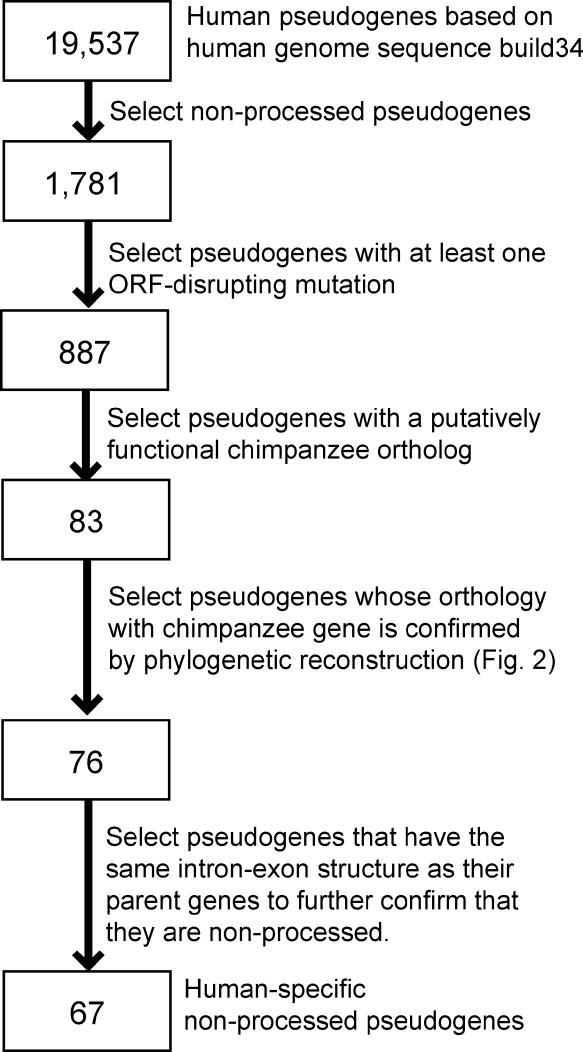
Flow Chart for Identifying the 67 Human-Specific Nonprocessed Pseudogenes The number of pseudogenes left after each step is given in the boxes.

**Figure 2:**
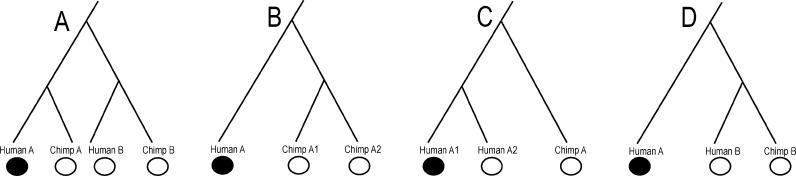
Evolutionary Scenarios for Human-Specific Pseudogenes and Non–Human-Specific Pseudogenes Functional genes and pseudogenes are represented by open and closed circles, respectively. A, A1, A2, and B represent hypothetical gene names. (A) The human-specific pseudogene has a functional chimpanzee ortholog. (B) The chimpanzee functional gene is most closely related to another chimpanzee functional gene. (C) The human pseudogene is most closely related to a functional human gene. (D) The chimpanzee functional ortholog is most closely related to a human functional gene. We consider (A) and (B) as human-specific pseudogenes.

We start from the 1,781 nonprocessed pseudogenes in the human pseudogene database [36], which contains 19,537 pseudogenes previously detected from the human genome sequence (Figure 1). Interestingly, the pseudogene database includes many sequences with complete ORFs, which are potentially functional genes but unannotated in the human genome sequence. These sequences are excluded from our analysis. We focus on the remaining 887 pseudogenes with at least one ORF-disrupting mutation, which may be a nonsense or frame-shift mutation. For each of the 887 pseudogenes, we conduct a BLAT search [39] in the draft chimpanzee genome sequence to identify the best chimpanzee hit, which is assumed to be the chimpanzee ortholog. Because most of the human pseudogenes were inactivated long before the human-chimpanzee divergence, only 83 human pseudogenes have putatively functional orthologs in chimpanzees. To ensure the orthology, we BLAT-search the human and chimpanzee genomes using the human pseudogene as a query and then take multiple top BLAT hits from both humans and chimpanzees to construct a phylogenetic tree. A human pseudogene is considered human specific when the tree in either Figure 2A or 2B is observed. That is, we exclude those cases where the human pseudogene is more closely related to a functional human gene (Figure 2C) or the putative chimpanzee ortholog is more closely related to a functional human gene (Figure 2D). This purging step left 76 human-specific pseudogenes. Finally, to verify that these pseudogenes are nonprocessed, we compare the genomic regions spanned by the intronless pseudogenes to the genomic regions spanned by their functional paralogs. If their functional paralogs have introns, we considered that the pseudogenes are actually processed pseudogenes but were misclassified previously. A total of 67 human-specific nonprocessed pseudogenes (Tables 1 and S1) are finally identified. While all pseudogenes with at least one ORF-disrupting mutation are examined, all detected human-specific pseudogenes contain either one (61 of 67 cases) or two mutations.

**Table 1:**
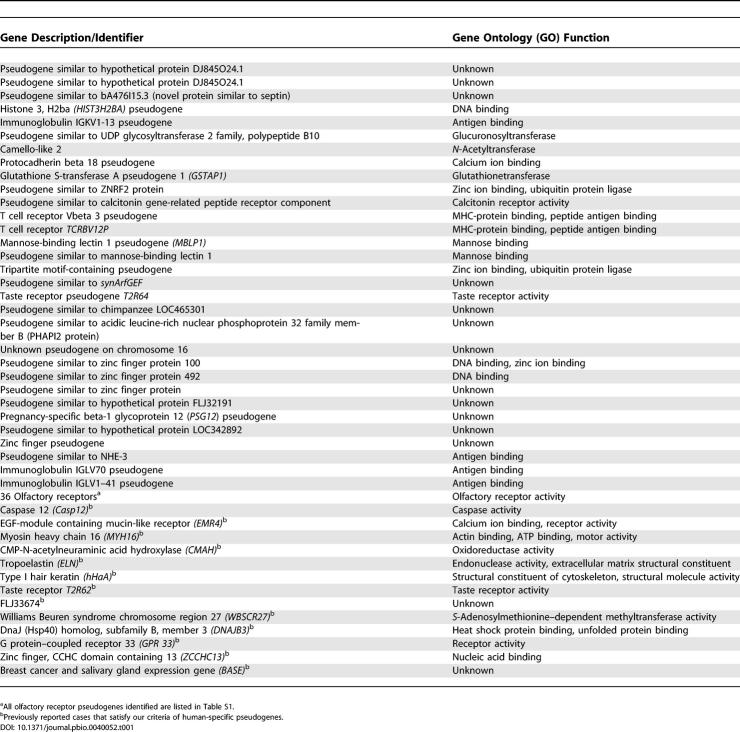
Human-Specific Pseudogenes Identified in This Study or Previously Reported

Because the human genome sequence was obtained from a small number of human individuals [40], it is possible that some human-specific pseudogenes we identified from the genome sequence have yet to be fixed in humans. In 2003, two large-scale studies verified that the dbSNP database at the National Center for Biotechnology Information covered 50% to 60% of all single nucleotide polymorphisms (SNPs) with frequencies greater than 10% [41,42]. Because the number of SNPs in dbSNP has more than tripled since these two studies, it is likely that the majority of SNPs with frequency greater than 10% are now covered in dbSNP. We used dbSNP to examine if the ORF-disrupting mutations in these pseudogenes are still segregating in humans and found that three of the 67 human-specific pseudogenes we identified are segregating with their functional alleles. They are immunoglobulin genes *IGKV1–13* and *IGLV1–41* and pregnancy-specific beta-1 glycoprotein 12 *(PSG12).* While no allele frequency data are available for the immunoglobulin genes, the null allele of *PSG12* is rare with a frequency of 0.7%.

### Functional Bias of Human-Specific Pseudogenes

Some of the pseudogenes we identified had been previously reported in the literature as human-specific pseudogenes. For example, one of the two previously identified human-specific bitter taste receptor pseudogenes [15,17] was identified with our method. Human-specific olfactory receptor (OR) pseudogenes have also been well documented [18,43,44], although the exact number is unknown. In a random sample of 50 human OR genes, Gilad et al. [18] found 12 to be human-specific pseudogenes. Extrapolated to the approximately 800 human ORs (HORDE database v.41) and considering the human OR pseudogene study excluded roughly 100 human pseudogenes from OR subfamily 7E, we would expect to identify 168 human-specific OR pseudogenes instead of only 36 that were identified with our method. Many reasons could account for this fivefold difference in the number of human-specific OR pseudogenes. First, Gilad et al.'s [18] study did not include polymorphism data, but population surveys have revealed that many human OR pseudogenes are still segregating with their functional alleles [45,46]. Therefore, the projected 168 human-specific OR pseudogenes is an overestimate. Second, because the OR gene family evolves via a rapid birth and death process [47], many OR genes were formed via species-specific duplication. Human pseudogenes in such species-specific duplications would fall into the category shown in Figure 2C and would not qualify as human-specific pseudogenes by our criteria. Finally, since OR genes have only one exon, their pseudogenes are often misclassified as intronless processed pseudogenes. Since we focused on nonprocessed pseudogenes, these misclassified pseudogenes would not be detected with our method.

Some human-specific pseudogenes previously reported in the literature, such as EGF-module containing mucin-like receptor *(EMR4)* [20], *MYH16* [23], *CMAH* [19], tropoelastin *(ELN)* [24], type I hair keratin *(*ϕ*hHaA)* [22], *CASP12* [33,34], and a bitter taste receptor *T2R62P* [15,17], were not detected with our method (Table 1), because they are all absent from the pseudogene database [36]. Additionally, our method did not detect the 15 pseudogenes identified in two earlier studies [29,30]. However, using our criteria aforementioned, only six of them qualify as human-specific nonprocessed pseudogenes (Table 1). The rest are either potentially processed, functional, or non–human specific or do not have complete sequence in the chimpanzee genome sequence. Thus, while our method does not reveal all human-specific pseudogenes, it has revealed substantially more than previous attempts [29,30]. Our method has been limited by the pseudogene database, and 1,781 [36] is likely a conservative estimate of the number of human nonprocessed pseudogenes. Our method has also been limited by the quality of the chimpanzee genome sequence as 198 of the 887 human pseudogenes had either no chimpanzee match or a chimpanzee match with incomplete sequence. It should be noted that in our analysis, pseudogenes are defined by the presence of premature stop codons or frame-shifting mutations. It is possible that a young pseudogene contains a severe mutation in its coding or regulatory regions but still retains its ORF. Such pseudogenes are undetectable with our method.

With these limitations, we analyzed a total of 80 human-specific pseudogenes identified here (67) and in previously studies (13). These pseudogenes have a diverse array of molecular functions (before pseudogenization), such as enzymes, receptors, and immunoglobulins (Table 2). To determine whether a molecular function is overrepresented, we used Fisher's exact test for each molecular function. The most striking bias is found in genes that function in chemoreception (olfaction and gustation) or immune response, two functional categories characteristic of rapidly evolving gene families with species-specific repertoires [6,47]. In particular, OR activity, a chemoreception function, is overwhelmingly overrepresented as nearly half of the identified pseudogenes are ORs, while less than 2% of human functional genes are ORs [47]. Additionally, major histocompatibility complex (MHC)-protein binding, mannose binding, antigen binding, and protein antigen binding, all involved in immune responses, are highly overrepresented. There are 14 identified pseudogenes with unknown functions. Of the 30 molecular functions present among the 80 human-specific pseudogenes, nine functions are significantly overrepresented (Table 2). In addition to the seven functions that can be largely included in chemoreception or immunity, there are two other functions that are overrepresented: calcitonin receptor activity and unknown functions. We also reexamined functional bias in our sample after excluding ORs and genes of unknown functions. Among the remaining 30 pseudogenes, ten molecular functions are significantly overrepresented, including taste reception, glucuronosyltransferase, calcitonin binding, caspase activity, glutathione transferase, *N*-acetyltransferase, and four immunity-related functions (Table 2). Note that glucuronosyltransferase activity and glutathione transferase activity are involved in detoxification; thus they may be grouped together with immunity genes as host-defense genes. Because multiple tests were conducted, Bonferroni corrections were applied. After the correction, chemoreception and immunity pseudogenes still remain significantly overrepresented (Table 2). Note that although a gene may be classified into more than one functional category, the above conclusion is not affected because chemoreception genes and immunity genes are mutually exclusive.

**Table 2:**
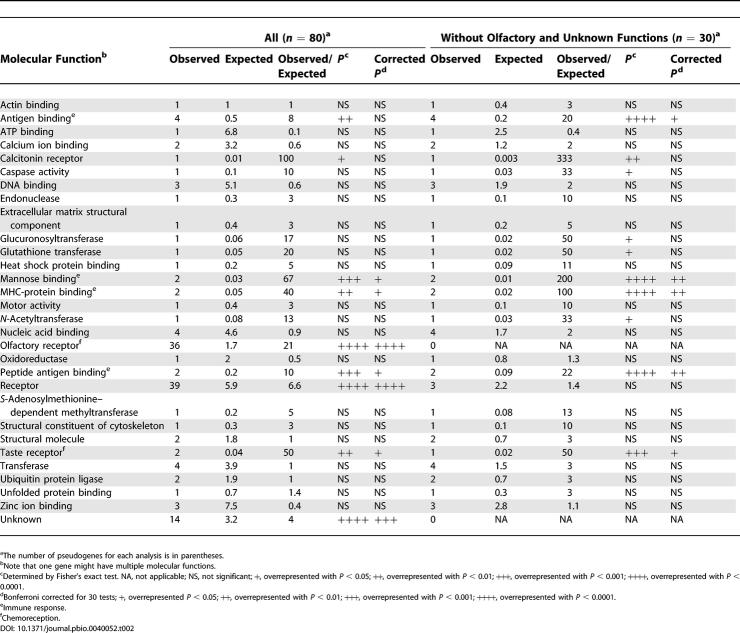
Functional Bias in Human-Specific Pseudogenes

### Adaptive Loss of *CASP12* in Human Evolution

Although the functional and physiological consequences of pseudogenization may be inferred from the gene ontology information (Table 1), a better understanding may be gained by examining the phenotypes of the mice with the functional orthologs deleted. However, among the 67 human-specific pseudogenes we identified, only one has such phenotypic information from mouse knockout experiments. The mouse gene is *Mbl1,* which is the ortholog of the human mannose-binding lectin 1 pseudogene. Interestingly, compared to the wild-type controls, mice homozygous for disruptions of *Mbl1* show increased survival due to lowered susceptibility to sepsis [48]. More interestingly, the primate mannose binding lectin 1 *(MBL1)* gene was duplicated in the common ancestor of humans and rhesus monkeys. After duplication, both daughter genes remain functional in rhesus monkeys and chimpanzees, but both became pseudogenes in humans. Because deletion of *Mbl1* increases survival in a mouse model of acute septic peritonitis, the losses of the two *MBL1* genes in humans may have been adaptive. But this hypothesis is difficult to test, as the fixations of the null alleles presumably occurred in the past 6 to 7 million years, most likely too long for traces of selective sweeps to be detected today. Nevertheless, the evolutionary comparison illustrates the possibility that some human-specific gene losses may have been driven by natural selection. The connection with sepsis in the above example prompted us to examine *CASP12,* another human-specific pseudogene related to sepsis. Because the pseudogenization of *CASP12* has yet to be complete [34], there is a high chance to detect the evolutionary forces responsible for the pseudogenization.

CASP12 belongs to the caspase family, which are cysteinyl aspartate proteinases that play important roles in the processing of inflammatory cytokines and the initiation and execution of apoptosis [49,50]. In humans, 11 functional caspase genes are known: *CASPASE1* through *CASPASE10* and *CASPASE14*. Human *CASP12* was identified as a pseudogene following the cloning of mouse *Caspase12* [33]. Compared with other mammalian orthologs, human *CASP12* contains a premature stop codon due to a C → T nonsense mutation at nucleotide position 629 of exon 4 [33,34]. This mutation leads to the production of truncated nonfunctional CASP12 in humans [34]. The null T allele is fixed in a sample of 347 non-Africans and has a frequency of 89% in 776 individuals of African descent [34]. Interestingly, the T allele is associated with a reduced incidence and mortality of severe sepsis [34], suggesting that the loss of functional CASP12 is beneficial to present-day humans. To test whether the nearly complete fixation of the null allele at *CASP12* has been driven by positive selection, we looked for signals of recent (incomplete) selective sweeps by examining the intraspecific variation of putatively neutral regions surrounding the C/T polymorphism. The positive selection hypothesis predicts that the level of polymorphism in these regions is lower in the T allele than in the C allele, especially in the proximity of the C/T polymorphism, due to the hitchhiking effect [51]. Furthermore, the frequency distribution of the neutral polymorphisms in the T allele should deviate from the neutral expectation, generating negative values of Tajima's *D* [52] and Fay and Wu's *H* [53].

**Figure 3:**
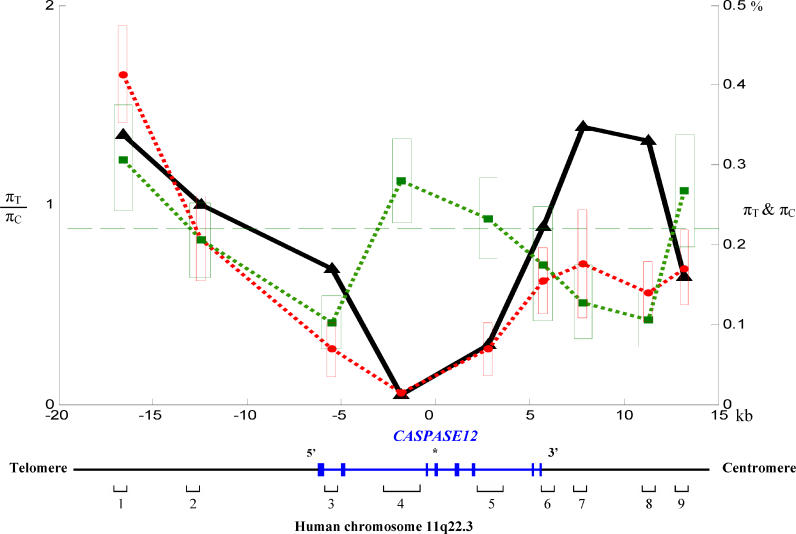
Intraspecific DNA Sequence Variation in Noncoding Regions Linked with the Human *CASP12* Gene *CASPASE12* is shown in blue, with the exons depicted by solid blue bars on the chromosome. The premature stop codon generated by the C → T nonsense mutation is shown by an asterisk in exon 4. The nine noncoding regions sequenced are indicated below the chromosome. Exons, introns, the nine noncoding regions, and spaces between regions are drawn to scale as indicated. Red circles (connected by the red dotted line) show nucleotide diversity per site among African T alleles (π_T_) and the red boxes shows π_T_ ± one standard error of π_T_. Green squares (connected by the green dotted line) show nucleotide diversity per site among African C alleles (π_C_) and the green boxes shows π_C_ ± one standard error of π_C_. The broken green line shows the mean π_C_ across the nine noncoding regions sequenced. Black triangles (connected by the black solid line) show the ratio between π_T_ and π_C_ for each region. π_C_ is estimated from eight alleles. π_T_ is estimated from 22 alleles for regions 4, 5, and 6 and from eight alleles for the other regions. When only eight alleles are used, π_T_ is 0.00018 ± 0.00007, 0.00129 ± 0.00071, and 0.00145 ± 0.00057 for regions 4, 5, and 6, respectively. π_T_ is significantly lower than π_C_ in regions 4 and 5 (Table S2).

From a sample of 63 humans of African descent, we identified four C/C homozygotes and 43 T/T homozygotes. We sequenced the four C/C homozygotes and four randomly chosen T/T homozygotes in nine noncoding regions of varying distances from the C/T polymorphism (Figure 3). The sequenced regions vary in size from about 600 to 2,400 nucleotides. In total, 53 and 29 SNPs were identified from 8,925 nucleotide sites in C/C and T/T individuals, respectively (Figure S1 and Table S2). Although the T allele is much more prevalent than the C allele in the population, the T allele has a significantly lower number of SNPs per nucleotide than the C allele in the linked regions (*P* < 0.01, Fisher's exact test). Nucleotide diversity per site (π) is also lower in the T alleles (π_T_ = 0.00131 ± 0.00019) than in the C alleles (π_C_ = 0.00218 ± 0.00031) (*P* = 0.02, two-tailed Z test). More strikingly, although the variation of π_C_ across the nine regions is more or less random, that of π_T_ exhibits a V shape, with the bottom of the valley located in region 4, which has its 3′ end only 607 nucleotides from the C/T polymorphism (Figure 3). When one moves approximately 10,000 nucleotides from this polymorphism, π_T_ rises to a level comparable to π_C_. To exclude the possibility that the low π_T_ observed around the C/T polymorphism was due to the use of a small sample, we sequenced seven additional T/T individuals of African descent in regions 4, 5, and 6. The π_T_ values obtained from the combined data of 11 individuals were either lower than or similar to those from the four individuals (Table S2), suggesting that the observation of low π_T_ is not due to a small sample. In region 4, where the greatest reduction in polymorphism is observed, only one SNP is found across the 2,413 nucleotide positions among the 22 T alleles sequenced. By contrast, 19 SNPs were found in the same region among eight C alleles examined. Region 4 was also sequenced in six non-Africans (all non-Africans are T/T homozygotes [34]), but no SNP was detected and all non-African T alleles are identical to the predominant T allele from Africans. This indicates a common origin of African and non-African T alleles.

In a formal test of the selective sweep hypothesis, we used coalescent simulations to examine whether the polymorphisms observed in region 4 can be explained by neutral models of evolution. Such tests require a sample that is representative of the population under investigation. We thus sequenced 20 additional African T/T homozygotes so that our sample comprises 89% (62 of 70) of T alleles and 11% (8 of 70) C alleles, expected in populations of African descent [34]. In the 70 chromosomes sequenced, the two most common haplotypes observed (with a total frequency of 61 of 70) are both from T alleles and these two haplotypes have only one nucleotide difference. Let *k*_1_ be the number of chromosomes with the most common haplotype in a sample and *k*_2_ be the number of chromosomes with the most frequent haplotype among those that are one nucleotide different from the most common haplotype in the sample. We first simulated the evolution of a population with a constant size. In 0.066% of the 50,000 replications, we observed *k*_1_ + *k*_2_ ≥ 61. We also simulated various demographic changes to mimic the evolution of human populations, and *k*_1_ *+ k*_2_ ≥ 61 was observed in fewer than 1% of simulation replications in all models considered (Table S3). These demographic models included ancient or recent population expansions, severe bottleneck, repeated bottlenecks with subsequent expansion, and population subdivision and admixture [54] (see Protocol S1). Previous studies suggested that the models used here are much more stringent than that associated with the real demographic history of humans [55,56]. Hence, our tests are conservative.

We also computed statistics *D* and *H* for regions 4 and 5 in the T allele, as these two regions have significantly lower π_T_ than π_C_ (Table S2). Both statistics were significantly negative in region 5 (*D* = −2.08, *P* < 0.01; *H* = −4.71, *P* < 0.025), consistent with the expectations from a selective sweep. *D* (−0.23, *P* = 0.47) and *H* (−0.90, *P* = 0.09) were not significantly negative in region 4, probably because the number of SNPs is too small for the statistic tests to be powerful. It should be noted that the above tests are less rigorous than the coalescent simulations because the tests are conducted on subsets of the genealogy [54]. Linkage disequilibrium (LD) can also be used to test recent selective sweeps if long-range haplotypes can be reliably inferred [57]. In the present case, however, long-range haplotypes are difficult to infer with certainty due to the small number of C/C homozygotes available. However, the genotypes shown in Figure S1 provide a visual indication of longer LD in T alleles than in C alleles and a decay of LD when one moves away from the C/T polymorphism, consistent with the recent origin of T alleles. Taken together, our observations, especially the proximity of the π_T_ valley to the C/T polymorphism and the coalescent simulations, strongly suggest that the spread of the T allele among Africans and non-Africans has been driven by positive selection and that the selective advantage was directly conferred by the C → T nonsense mutation.

### Dating the Pseudogenization Event and Selective Sweep in *CASP12*

When did the pseudogenization of human *CASP12* start? We took two approaches to estimate the age of the T allele. In the first method, we used the information of noncoding region 4, which is longest among the nine sequenced regions and is also closest to the C/T polymorphism. The founding haplotype of T alleles is inferred, and the proportion *(P)* of present-day T alleles identical to the founding haplotype is estimated. It can be shown that *P* = (1 − *r*)*^G^*, where *G* is the age of the T allele in generation and *r* is the total rate of mutation and recombination per sequence per generation [58]. In the present case, it is easy to infer the founding haplotype (for region 4) because of the low polymorphism and the availability of an outgroup (chimpanzee) sequence. *P* is estimated to be 0.811 based on the observation of 60 copies of the founding haplotype in a total of 74 T alleles sequenced (including both Africans and non-Africans). The mutation rate is estimated to be 23/(12 × 10^6^) × 25 = 4.792 × 10^−5^ per sequence per generation. Here 23 is the average number of nucleotide differences between human and chimpanzee in region 4, 12 × 10^6^ is twice the divergence time in year between the two species [31], and 25 is the average human generation time in years. The recombination rate is estimated to be 0.7 × 10^−8^ × 3,720 = 2.269 × 10^−5^ per sequence per generation, where 0.7 × 10^−8^ is the pedigree-based recombination rate per generation per nucleotide at the *CASP12* locus [59] and 3,720 is the number of nucleotides between the 5′ end of region 4 and the C/T polymorphism. We thus estimated that *G* = 2,970 generations (Figure S2A), which corresponds to 74,250 years. The 95% confidence interval for *P* is between 0.647 to 1. If we consider the sampling error of *P*, the 95% confidence interval for the estimated time is from 0 to 154 thousand years. The standard error of the estimated mutation rate is 1/√23 = 21% of the estimate, while the error of the recombination rate is difficult to evaluate.

In the second method, we used a deterministic selection model [60] to estimate the number of generations required for the T allele to rise to its present-day frequency among individuals of African descent. It has been estimated that the incidence of severe sepsis is *I* = 0.59% and the mortality rate is *M* = 26.5% among African Americans [61]. The genotype frequencies among individuals of African descent are *f*_C/C_ = 1.675%, *f*_C/T_ = 18.6%, and *f*_T/T_ = 79.77%, respectively [34]. Here we used the genotype frequency data from [34] because their sample is considerably larger than ours. The proportions of the three genotypes among severe sepsis patients have been estimated to be *P*(C/C|sepsis) = 10.5%, *P*(C/T|sepsis) = 29.0%, and *P*(T/T|sepsis) = 60.5% [34]. Using Bayes theorem, we calculated the survival rate *(S)* for a given genotype X by *S*_X_ = 1 − *MP*(sepsis|X) = 1 − *IMP*(X|sepsis)/*f*_X_ and obtained *S*_C/C_ = 0.9902, *S*_C/T_ = 0.9976, and *S*_T/T_ = 0.9988. Here we assumed that the prereproductive-age incidence of sepsis in much of the human history is comparable to the total incidence of sepsis estimated today [61]. The relative fitness of C/C to the fitness of T/T is therefore *W*_C/C_ = *S*_C/C_/*S*_T/T_ = 0.991. Similarly, *W*_C/T_ = *S*_C/T_/*S*_T/T_ = 0.999 and *W*_T/T_ = 1. The selective disadvantage of C/C compared with T/T is *s* = 1 − *W*_C/C_ = 0.009 and the degree of dominance of the C allele relative to the T allele is *h* = (1 − *W*_C/T_)/(1 − *W*_C/C_) = 0.11. The number of generations required for a given change in allele frequency was calculated using the differential equation *dp*/*dt* = *p*(1 − *p*)*s*[*ph*+(1 − *p*)(1 − *h*)] with the current T frequency *p* = 0.891 [34] and the initial T frequency *p*_0_ = 1/(2*N*), where *N* is the effective population size of humans [60]. The calculated number of generations is *t* = 2,111 (Figure S2B), under the assumption of an effective population size of 10^4^ individuals [62,63]. In this computation, we ignored the effect of random genetic drift because 2*Ns* = 180 ⋙ 1 and the behavior of the alleles is dominated by selection [64]. Because of the sampling error, the 95% confidence interval of *p* is [0.875, 0.907], which gave the 95% confidence interval of the time required for the T allele to reach today's frequency to be 51,000 to 55,000 years. Note that the actual error of the time estimate may be considerably larger because the estimation errors of *h* and *s* are difficult to assess. Here we assumed that positive selection acted as soon as the null allele appeared. It is possible that the null allele was initially neutral but later became beneficial due to a change in the genetic or environmental background. If this is the case, the appearance of the T allele would be earlier than dated by this method.

Strictly speaking, the first approach we used was to date the appearance of the T allele, whereas the second approach was to date the onset of the selective sweep. These two events were not necessarily simultaneous, although the appearance of the T allele was a prerequisite for the selective sweep. Despite the potentially large errors, the two estimates were close, suggesting that the T allele might have been beneficial since its appearance. Because the T alleles of Africans and non-Africans share the same origin, the C → T nonsense mutation must predate the out-of-Africa migration of modern humans, which is believed to have occurred 40,000 to 60,000 years ago [65]. Our dating suggests that the pseudogenization of *CASP12* began not long before this migration. As a comparison, it is interesting to compute the mean time required for a neutral allele to rise to the current frequency of *P* = 0.891. This can be estimated by −4*Np*(ln*p*)/(1 − *p*) = 37,736 generations, or 943,000 years [66]. In the above, *N* is the effective population size of humans and is assumed to be 10^4^. Thus, it would have taken a considerably longer time for the null allele to reach today's frequency if it were neutral.

### Implications

The identification of the human-specific gene losses helps us understand the human-specific features and their genetic basis. The overwhelming overrepresentation of chemoreception and immunity functions among the human-specific pseudogenes indicates substantive changes in these two aspects of physiology during human evolution. The loss of chemoreception genes is broadly consistent with the common belief that humans have a reduced sense of smell (but see [67]) and may reflect significant changes in the way humans interact with each other and with the environment, human diet, and human behavior during the past few million years [15,18]. The losses of many immunity genes are consistent with and may in part account for the many differences between humans and their related primates in susceptibility to various pathogens such as HIV/simian immunodeficiency virus and Plasmodium falciparum (malaria). As aforementioned, the species-specific losses of several other genes such as *CMAH* and *MYH16* have been suggested to be responsible for certain human-unique features or related to human adaptations. Our identification of human-specific pseudogenes opens the door for systematic evaluations of the timings, functional consequences, and potential roles of gene loss during human evolution.

Identifying human-specific pseudogenes is only one half of the story. It is unclear whether the functional bias we observed in human-specific pseudogenes is also found among chimpanzee-specific pseudogenes. Unfortunately, computational identification of chimpanzee-specific pseudogenes requires a highly accurate chimpanzee genome sequence [6], because a small sequencing error, such as a misreading of trinucleotide GGG into GG in a coding sequence, causes frame-shifting and produces erroneous species-specific pseudogenes. Our preliminary analysis reveals many more chimpanzee-specific pseudogenes than human-specific pseudogenes, but subsequent resequencing of a few chimpanzee “pseudogenes” suggests that this difference in pseudogene number is likely due to errors in the currently available chimpanzee genome sequence, which has a low accuracy (3.5× coverage). Similarly, although 53 potentially chimpanzee-specific gene losses were identified in a recent analysis, the majority of them could not be confirmed [6]. These uncertainties notwithstanding, detailed analyses of olfactory and bitter taste receptor genes suggested that the pseudogenization rates in these chemoreception genes are lower in chimpanzees than in humans [15,17,18] (but see [16]). Thus, it is expected that the two lineages have differences in the pattern of pseudogenization.

Our population genetic study provided strong evidence that the nearly complete fixation of a null allele at human *CASP12* has been driven by positive selection, possibly because it confers resistance to severe sepsis. *CASP12* is a functional gene in all mammals surveyed except humans [34], suggesting that it is indispensable in a typical mammal. The functional human CASP12 acts as a dominant-negative regulator of essential cellular responses including the necrosis factor-κB and interleukin-1 pathways; it attenuates the inflammatory and innate immune response to endotoxins [34]. Because an appropriate level of immune response that is neither excessive nor insufficient is important to an organism, one can imagine that the immune suppression function of CASP12 becomes harmful when the immune system cannot fully respond to a challenge. It is likely that during human evolution alterations in our genetic and/or environmental background resulted in a malfunction of the immune response to endotoxins, which rendered the previously necessary function of CASP12 deleterious in humans and the null allele advantageous over the functional one. Identification of such genetic and/or environmental alterations will be valuable for understating human-specific immune functions. It is interesting to note that mouse Caspase12 is implicated in amyloid-induced neuronal apoptosis, whereas the functional form of human CASP12 does not have this function. The reasons and consequences of this difference, particularly in relation to the human-specific pathology of Alzheimer disease, are intriguing [6].

The “less-is-more” hypothesis emphasized that gene loss can sometimes play an active role in evolution [11], with the premise that gene loss may provide opportunities for future adaptations. Our finding that gene loss itself can be adaptive supports and extends the “less-is-more” hypothesis. Although *CASP12* is the first demonstrated case of adaptive gene loss in humans, similar events may have occurred or are occurring at other loci, possibly including the human-specific pseudogenes we identified, because human lifestyle and environmental interactions have changed immensely in the past few million years. Such changes may have made formerly useful gene functions harmful. Pseudogenizations of the two paralogous *MBL1* genes in humans and the finding that deleting *Mbl1* increases survival in a mouse model of sepsis suggests that the losses of the two human *MBL1* genes may have also been driven by positive selection. The common connection to sepsis among *CASP12* and the two *MBL1* genes reinforces the conjecture that the way humans respond to sepsis and/or the threat of sepsis to humans might have been significantly different from those in other species. In the context of pathogenic threats, it is interesting to mention two examples where human null alleles are selected for in certain geographic areas [58,68]. In the first example, a null allele generated by a 32-nucleotide deletion in the chemokine (C-C motif) receptor 5 *(CCR5)* gene was subject to positive selection in Caucasians in the recent human history [58] (but see [69]). *CCR5* is used by pathogens, such as HIV, as a coreceptor to enter host cells; the null allele protects humans from attacks of these pathogens. The exact pathogens that were responsible for the spread of the *CCR5*-null allele, however, are still under debate [70]. In the second example, a null allele at the Duffy blood group locus was shown to be beneficial in some Africans, probably because it confers resistance to malaria [68]. Nevertheless, in both of these examples, the null alleles appear to be less fit than the functional alleles when the pathogens are rare or absent. Thus, the positive selection for the null alleles is limited to small geographic areas, and it is unlikely that they will lead to the eventual loss of the two human genes. By contrast, *CASP12* has been lost in non-Africans and is nearly lost in Africans.

How often does adaptive gene loss occur in general? While this problem has not been investigated systematically, two nonhuman cases have been reported recently. The first occurred in a gene responsible for pheromone synthesis in insects, and the pseudogenization led to the origin of a partially reproductively isolated race of Drosophila melanogaster [71,72]. The second case involves a gene whose functional product prevents selfing in plants and the pseudogenization event allowed the evolution of self-pollination in Arabidopsis thaliana [73]. Given the high frequency of pseudogenization in eukaryotic genomes, one may speculate that adaptive gene loss is not uncommon. Interestingly, two of the three adaptive pseudogenizations so far documented happened to genes that are involved in chemoreception or immunity, consistent with the previous finding that genes of these functions tend to evolve rapidly with high rates of turnover [74,75] and our current finding that these functions are overrepresented among human-specific pseudogenes. Although detection of adaptive gene loss is restricted due to a rapid decay of population genetic signals of selective sweeps [32], it is possible that adaptive gene loss is more frequent than previously thought, especially from the above two functional categories. This said, the study of the roles that gene losses play in evolution has just begun; more empirical evidence is needed to demonstrate the importance of the “less-is-more” hypothesis during evolution in general and human evolution in particular.

## Materials and Methods

### Human-specific pseudogenes

The human pseudogene database includes pseudogenes detected from the human genome build34 and can be found at http://www.bork.embl-heidelberg.de/Docu/Human_Pseudogenes. We restricted our search to nonprocessed pseudogenes. Surprisingly, half of the nonprocessed pseudogenes from the database had neither a nonsense nor a frame-shifting mutation. These are potentially functional genes, but unannotated in the human genome sequence at the time of building the pseudogene database. We only investigated pseudogenes with at least one ORF-disrupting mutation. We BLAT-searched [39] the identified human-specific pseudogenes against the chimpanzee genome sequence from the UCSC Genome Browser (http://genome.ucsc.edu) and identified top hits based on 95% or greater nucleotide identity in the coding region and correct synteny. We BLASTed (basic local alignment search tool) the identified human-specific pseudogenes against the nonredundant NCBI Human Database (http://www.ncbi.nlm.nih.gov, identified their best-hit functional genes, and retrieved their molecular functions from the Gene Ontology database (http://www.geneontology.org). Gene trees were reconstructed using the neighbor-joining method [76] by MEGA3 [77].

Only five of the 67 newly identified human-specific pseudogenes have more than two exons. To determine if these pseudogenes could be alternatively spliced to yield functional proteins, we looked for splice variants of the best-hit human functional paralog in Ensembl (www.ensembl.org) and none of them have known alternative splicing. To determine if there is a bias for any of the functional categories, we computed the expected number of pseudogenes for a given functional category by 80*m*/26445, where *m* is the number of functional genes belonging to the functional category and was obtained from Gene Ontology database annotations (http://www.geneontology.org/GO.current.annotations.shtml, 26,445 is the total number of human functional genes and 80 is the total number of human-specific pseudogenes (identified in this and previous studies). Fisher's exact tests were then conducted to compare the observed and expected numbers of human-specific pseudogenes for given functional categories. We retrieved from the mouse genome informatics web site (http://www.informatics.jax.org) the mouse knock-out phenotypes for the 67 human-specific pseudogenes we identified. The duplication and evolution of the primate *MBL1* genes were analyzed using the human and chimpanzee genome sequences, as well as the rhesus monkey genome sequence assembly (http://genome.ucsc.edu).

### DNA amplification and sequencing of *CASP12* alleles

All human genomic DNA samples were purchased from Coriell Cell Repository (http://locus.umdnj.edu/nigms). The genotypes of 63 individuals of African descent at the C/T polymorphism (position 629 of exon 4) were determined by sequencing a portion of exon 4. These individuals included 48 African Americans, six African pygmies, and nine Africans (south of the Sahara). Four C/C homozygotes (three African Americans and one African pygmy), 43 T/T homozygotes, and 16 C/T heterozygotes were identified. The T allele has a frequency of 81% ± 0.035% in our sample, slightly lower than that (89%) reported in a previous study, which was based on a much larger sample [34]. All four C/C individuals and four randomly picked T/T individuals (three African Americans and one African pygmy) were sequenced in nine noncoding regions as shown in Figure 3. To ensure that the low polymorphism found among T/T individuals in regions 4, 5, and 6 was not due to the small sample size, we sequenced seven additional T/T individuals (all African Americans) in the three regions. The genotypes of six non-Africans (two Caucasians, one Chinese, two Pacific Islanders, and one Andes) at the C/T polymorphism were also determined by the same approach and all were found to be T/T homozygotes. (Note that the fixation of the T allele in non-Africans was previously demonstrated in a sample of 347 individuals [34].) Region 4 was sequenced in these six non-Africans and no SNPs were found. For conducting coalescent simulations by the ms program [78], we sequenced 20 additional T/T individuals of the African descent for region 4, so that our sample of Africans comprised four C/C and 31 T/T individuals, with the frequency of T alleles being 89%, which is expected for Africans [34]. Our sample can be treated as a random sample under the reasonable assumption of random mating with respect to the C/T polymorphism.

The experimental procedure was as follows. Fragment-specific primers were designed according to the human genome sequence. PCRs were performed with MasterTaq (Eppendorf, Hamburg, Germany) under conditions recommended by the manufacturer. PCR products were separated on 1.5% agarose gel and purified using the Gel Extraction Kit (Qiagen, Valencia, California, United States). Amplified DNA fragments were sequenced from both directions in an automated DNA sequencer using the dideoxy chain termination method. Sequencher (Gene Codes, Ann Arbor, Michigan, United States) was used to assemble the sequences and to identify DNA polymorphisms. All singletons were confirmed by an independent PCR and sequencing experiment. After removing the primer regions, each sequenced fragment is 500 to 800 nucleotides long. All the SNPs identified in this study are listed in Table S4.

### Population genetic analysis

Nucleotide diversity per site π [52], Tajima's *D* [52], and Fay and Wu's *H* [53] were computed by DnaSP [79]. Gaps in the sequence alignments were excluded from the analysis. The chimpanzee genome sequence available in GenBank was used as an outgroup in computing *H*. Tajima's test [52] and Fay and Wu's test [53] were conducted by DnaSP using coalescent simulations with 50,000 replications under the assumption of no recombination, which gave more conservative results than when recombination is considered. To test the hypothesis of selective sweeps more rigorously, we modeled various demographic scenarios of human populations by coalescent simulations (50,000 replications per model). The parameters used in the coalescent simulations are described in Protocol S1. Two methods were used to estimate the age of the T allele, as described in detail in Results/Discussion.

## Supporting Information

Figure S1 Genotypes of the Four C/C Homozygotes and Four T/T Homozygotes That Were Sequenced in All Nine Noncoding Regions Each row represents one human individual and each column represents one SNP site. The top four individuals are homozygous for the functional *CASP12* allele, and the bottom four individuals are homozygous for the null allele. Blue, yellow, and green squares indicate homozygotes for the ancestral allele, homozygotes for the derived allele, and heterozygotes, respectively, at each SNP site. The nucleotide position of each SNP site is given at the bottom of the figure with the ancestral/derived nucleotides indicated. The nucleotide positions are relative to the start codon ATG. On the top of the figure is the chromosome, with the exons of *CASP12* depicted by solid blue bars on the chromosome. The premature stop codon generated by the C → T nonsense mutation is shown by an asterisk in exon 4. The nine noncoding regions sequenced are indicated below the chromosome.
(64 KB PDF).

Figure S2 Estimating the Age of the Null Allele and the Onset of the Selective Sweep (A) Decline of the frequency (*P*) of the founding haplotype of the null allele over generations *(G).* We used the formula *P* = (1 − *r*)*^G^*, with the sum of the mutation and recombination rate *r* being 7.061 × 10^−5^ per generation. The dashed line shows the estimated *P* at present and its corresponding *G*.
(B) The increase of the frequency *(p)* of the null allele over generations *(t)* by positive selection, based on the differential equation *dp*/*dt* = *p*(1 − *p*)*s*[*ph* + (1 − *p*)(1 − *h*)]. Here we used *p*_0_ = 0.00005, *h* = 0.11, *s* = 0.009. The dashed line shows the estimated *p* at present and its corresponding *t*.
(171 KB PDF).

Protocol S1 Supplementary Methods (79 KB PDF).

Table S1 Human-Specific Pseudogenes (25 KB XLS).

Table S2 Intraspecific Variations in Nine Noncoding Regions Linked to Human *CASP12*
(19 KB XLS).

Table S3 Results from Coalescent Simulations (17 KB XLS).

Table S4 SNPs Identified in the Noncoding Regions Linked with *CASP12*
(20 KB XLS).

## Abbreviations

BLAST: basic local alignment search tool
BLAT: BLAST-like alignment tool
CASP12: CASPASE12
CCR5: chemokine (C-C motif) receptor 5
CMAH: CMP-*N*-acetylneuraminic acid hydroxylase
HIV: human immunodeficiency virus
LD: linkage disequilibrium
MBL1: mannose binding lectin 1
MHC: major histocompatibility complex
MYH16: masticatory myosin heavy chain 16
OR: olfactory receptor
PSG12: pregnancy-specific beta-1 glycoprotein 12
SNP: single nucleotide polymorphism
