# Supplementary material for: Gene Losses during Human Origins
Source: PLoS Biol. 2006 Feb 14;4(3):e52. doi: 10.1371/journal.pbio.0040052 (PMC1361800; doi:10.1371/journal.pbio.0040052)
Supplement: Supplementary file 1 [file pbio.0040052.sd001.pdf]

Supplementary Methods

Our sample for region 4 included 4 C/C and 31 T/T individuals of African descent. Thus, the sample frequency of the T allele is 89%, same as the expected value for humans of African descent [34]. In the 70 chromosomes sequenced, 19 SNPs were found. The two most common haplotypes observed (with a total frequency of 61/70) are from T alleles and these two haplotypes have only one nucleotide difference. Let k_1 be the number of copies of the most common haplotype in a sample and k_2 be the number of copies of the most frequent haplotype among those that are one nucleotide different from the most common haplotype in the sample. In coalescent simulations, we calculated the proportion of cases (out of 50,000 replications) in which $k_1+k_2 \geq 61$. We used the ms program [78] to generate independent samples under a variety of neutral models with different demographic histories. The number of alleles and the number of segregating sites were set to be 70 and 19, respectively. The pedigree-based recombination rate of $R=0.7\times10^{-8}$ per generation per nucleotide at the *CASP12* locus [59] was used. We examined 9 demographic models following Evans and colleagues [54]. We assumed an effective population size of $N=10^4$ [62, 63], as the genetic variants of non-Africans are usually subsets of those of Africans. In the following command lines, 2413 is the length of region 4 in nucleotide, and 0.675 is the population recombination rate for region 4 when $N=10^4$ is used ($\rho=4NR=4\times10^4\times0.7\times10^{-8}\times2413=0.675$).

The 9 models tested and the command lines used in the ms program are:

1) Constant population with an effective size of 10^4,

```
./ms 70 50000 -s 19 -r 0.675 2413
```

2) An ancient population expansion from 10^4 at 5,000 generations ago exponentially to 10^7 today,

```
./ms 70 50000 -s 19 -r 675 2413 -G 55262.04223 -eG 0.000125 0
```

3) A recent population expansion from 10^4 at 1,000 generations ago exponentially to 10^7 today,

```
./ms 70 50000 -s 19 -r 675 2413 -G 276310.2112 -eG 0.000025
```

4) A severe bottle neck starting 5,000 generations ago that reduced the population from 10^4 instantly to 10^3 and lasted until 2,500 generations ago at which point the population started to expand exponentially to 10^7 today,

```
./ms 70 50000 -s 19 -r 675 2413 -G 147365.446 -eG 0.0000625 0 -eN 0.000125 0.001
```

5) Repeated bottlenecks for five successive rounds starting 7000 generations ago, each from 10^4 instantly to 10^3 for 500 generations followed by exponential recovery back to 10^4 over another 500 generations, except at the end of the fifth bottleneck 2500 generations ago which was followed by exponential growth to 10^7 today,

```
./ms 70 50000 -s 19 -r 675 2413 -G 147365.446 -eG 0.0000625 0 -eN 0.000075 0.001 -eG 0.000075 184206.8074 -eG 0.0000875 0 -eN 0.0001 0.001 -eG 0.0001 184206.8074 -eG 0.0001125 0 -eN 0.000125 0.001 -eG 0.000125 184206.8074 -eG 0.0001375 0 -eN 0.00015 0.001 -eG 0.00015 184206.8074 -eG 0.0001625 0 -eN 0.000175 0.001
```

6) Population structure where the initial 70 chromosomes were split equally into 2 different subpopulations under constant population size with 1 migration per generation,

```
./ms 70 50000 -s 19 -r 0.675 2413 -es 0.0 1 0.5 -eM 0.0 1.0
```

7) Population structure where the initial 70 chromosomes were split equally into 3 different subpopulations with 1 migration per generation,

```
./ms 70 50000 -s 19 -r 0.675 2413 -es 0.0 1 0.3333 -es 0.0 1 0.5 -eM 0.0 1.0
```

8) Population structure where the initial 70 chromosomes were split equally into 4 different subpopulations with 1 migration per generation,

```
./ms 70 50000 -s 19 -r 0.675 2413 -es 0.0 1 0.25 -es 0.0 1 0.333 -es 0.0 1 0.5 -eM 0.0 1.0
```

9) Population structure where the initial 70 chromosomes were split equally into 5 different subpopulations with 1 migration per generation,

```
./ms 70 50000 -s 19 -r 0.675 2413 -es 0.0 1 0.2 -es 0.0 1 0.25 -es 0.0 1 0.333 -es 0.0 1 0.5 -eM 0.0 1.0
```
