# Supplementary material for: Gene Losses during Human Origins
Source: PLoS Biol. 2006 Feb 14;4(3):e52. doi: 10.1371/journal.pbio.0040052 (PMC1361800; doi:10.1371/journal.pbio.0040052)
Supplement: Supplementary file 2 [file pbio.0040052.sg001.pdf]

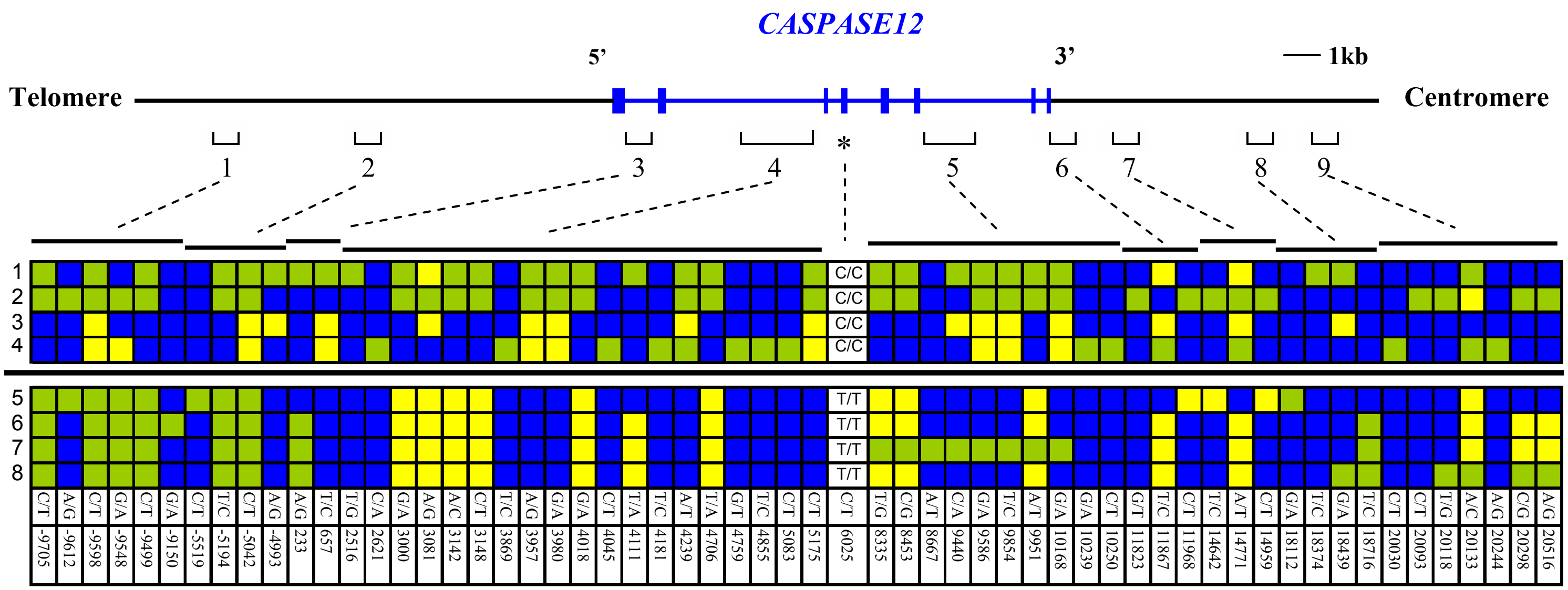

Supplementary Fig. 1
